# HEALTHY DIETARY PRACTICE AND SELF-RATED HEALTH AMONG KOREAN OLDER ADULTS: THE MEDIATING ROLE OF PHYSICAL ACTIVITY

**DOI:** 10.1093/geroni/igae098.3850

**Published:** 2024-12-31

**Authors:** Joosuk Chae, Hyunmi Kim, In-Taek Lim

**Affiliations:** SCELA (Comprehensive Support Center for the Elderly Living Alone), Seoul, Republic of Korea; SCELA (Comprehensive Support Center for the Elderly Living Alone), Seoul, Republic of Korea; Catholic University, Seoul, Republic of Korea

## Abstract

This study explored the relationship between healthy eating habits, physical activity, and self-rated health perception among older Korean adults, with a specific focus on whether physical activity level, as measured by the Global Physical Activity Questionnaire (GPAQ), serves as a mediating factor. We examined whether healthy eating habits influenced self-rated health perceptions through increased physical activity using Process Macro 4.3. The analysis included 1,468 older adults aged ≥65 years from the first year (2022) of the 9th Korea National Health and Nutrition Examination Survey (KNHANES), a nationally representative panel survey conducted in Korea from 2022 to 2024. The findings confirmed that healthy eating habits affected self-rated health perceptions via increased physical activity. This suggests that healthy eating habits positively help increase physical activity, which in turn enhances self-rated health status. These results highlight the importance of an integrated approach that incorporates healthy eating education and physical activity promotion when developing future care service plans for older adults in Korea. Based on these findings, more comprehensive guidelines can be established for social workers to design individualized programs within the older adults’ care service delivery system.

